# Buffalo Obstetrical Society

**Published:** 1891-05

**Authors:** Wm. C. Krauss

**Affiliations:** Secretary; 473 Virginia Street


### BUFFALO OBSTETRICAL SOCIETY.

Reported by WM. C. KRAUSS M. D., Secretary.

*Regular Meeting, held February 24, 1891.*

The President, Dr. A. H. Briggs, in the chair.

The paper of the evening, entitled Varicose Veins, was read by Dr. Alphonse Dagenais.

In selecting varix and its consequences, I am aware of the difficulties of treating a subject to which authors on diseases of women attach very little importance. They seem to think this subject of not sufficient gravity to give it lengthy discussion. It is my object to confine this dissertation to the consideration of varices of the lower extremities, which are oftener met in general practice, and particularly in the pregnant female. Surgery claims the right almost exclusively of treating the subject, which, "au premier abord," seems to belong to it. But, in its progressive and wonderful march in the path of science, surgery wishes to control everything. Therefore all gynecologists are surgeons, and in so saying we only give another proof of the great affinity existing among other different branches of the medical sciences.

The cases which come under observation (with a few exceptions occurring among men) seem to call for a separate consideration in the diseases of women, and for that very reason I wish to hear the different views entertained by the members of this Society.

Permit me a short anatomical description of the veins. We all know their starting point from minute radioles in the capillaries, which are found everywhere in the textures of the body, that converge to constitute larger and larger branches, till they terminate in the main trunks which convey the venous blood directly to the heart. They are provided with three coats like the arteries, but are thinner, and have more frequent communications between themselves, this being strikingly exhibited in the frequent inosculations of the spinal veins, as well as in the sepermatic and vesical plexuses. The benefit of these inosculations is very apparent, preventing to a certain extent the obstructions to which the veins are liable from their inability to overcome much impediment by the force of their current. We know, too, that the blood current is maintained by the slight force, *vis a tergo*, remaining of the contraction of the left ventricle and by muscular pressure.

One reads in Kirke's Physiology, that—

The great obstacle to the upward current of the blood in the veins of the trunks and extremities in the erect position, supposed to be presented by the gravitation of the blood, has no real existence, so long as both arteries and veins contain a continuous column of blood. The force of gravitation, whatever be the position of the body, can have no power to remove or resist the motion of any part of the blood in any direction. The lowest blood-vessels have, of course, to bear the greatest amount of pressure; the pressure on each part being directly proportionate to the height of the column of blood above it, hence their liability to distension. But this pressure bears equally on both arteries and veins, and cannot either move or resist the motion of the fluids they contain, so long as the columns of fluid are of equal height in both and contiguous

I have quoted the above in order to show that external causes must exist to produce varix; for the laws of equilibrium are observed with absolute correctness, in the dispositions of the fluid contained in the blood-vessels.

There are two causes for varix: First, accidental and mechanical, of which pregnancy and confinement are the main factors. Second, a general laxity of the tissues which predisposes to it and in which cases the normal muscular pressure is missing; hence this tendency to enlarge.

Even without mechanical interference in such cases, the coating of the veins themselves must share in this laxity; although absent in younger years, they are developed chiefly during the active middle period of life; still their appearance may be postponed until old age. Obstruction to the circulation is, we know, the passive cause of their formation; it is the reason why they coincide with certain diseases of the heart, lungs, or liver, because such a circulation of the blood suffering from such a disease is easily influenced, and this influence is more prevalent upon the veins of the legs, because the pressure of the column of blood which these vessels have to resist is increased by gravitation. So, if there is a tendency to dilatation by obstruction, such force, instead of moving the blood column, is expended upon the vein walls. In persons with relaxed and debilitated veins, large varices will sometimes appear suddenly after violent muscular exercise.

The anatomical changes in varicose veins have been described by Andral, who says that the vessels may become thickened or thinned; they may also become lengthened, so that the veins are rendered tortuous; that the dilatation may be unequal; that varix of the femoral vein, which requires to be distinguished from a femoral hernia, is formed by the yielding of the vessel at its junction with the internal circumflex, saphena and profunda veins. The valves of the veins are not destroyed by the backward pressure of the column of the blood, but they are flattened against the vein walls and often shrink and become atrophied. The tissues surrounding the diseased veins become gradually absorbed as a result of the continued pressure of the varix, and in cases of long standing we often find the surrounding tissue thickened and hard from simple edema.

More care should be given to the first symptoms of this troublesome malady. When a patient first applies for relief, instructions must be given as to the importance of care, and warning should be served of the ultimate liability to the formation of varicose ulcers. But patients seldom have the inclination or the opportunity to submit to rigid treatment for the cure of a disease which causes comparatively little inconvenience, and when this is the case simple compression of the distended veins will often give great relief and even prevent the disease from growing worse. It is useless for me to describe the symptoms of varix with which all are familiar. Suffice it to mention that statistics obtained by Verneil at the Hôtel Dieu, show that one limb is diseased just as often as the other—in fact, the cases are rare in which the disorder does not affect both limbs simultaneously. The best authorities, says Holmes, admit that the treatment of varices of long standing are inveterate, for although much good may be done for their relief they cannot be cured.

But I think if more care was taken of the general health better results would be obtained. The feeble and ill-nourished patient must be invigorated by tonics and a generous diet. The plethoric with an embarrassed portal circulation will be benefited by repeated doses of purgative medicine and saline baths; some special symptoms demand special care. When there exists an acquired laxity of the veins, tinct. hamamelis, ergot, and above all tinct. ferri mur. give good results; it is also advisable that the legs should be rested in the horizontal or elevated position, and the limb thus rested should be bandaged from the toes to the knee. The pressure should be firm and equal and the bandage renewed daily. At the time of the renewal of the bandage, rubbing with the hand should be advised, for at least a quarter of an hour, as, by this treatment, the circulation through the subcutaneous veins is rendered brisk and the tonicity of the walls reëstablished. With this rest of the muscles, the improved tone of the superficial vessels, and the improvement of the general health, a good result may be anticipated. As to bandages, the elastic spiral bandage or the elastic webb stocking are the most convenient and afford the necessary support; they should be removed at night and replaced in the morning.

The most difficult to manage are old varices surrounded by a quantity of indurated tissue, which protects them from ordinary pressure. Harapath suggests the division of the fascia lata at the saphenous opening to relieve the pressure on the femoral veins. Of the different operations suggested, objections have been made to the peril to the patient's life from diffuse inflammation extending along the course of the vein which had been operated on; but cases for operation must be selected with care. Celsus draws a distinction between straight and convoluted varices; for the first, he recommends that they should be exposed by cutting through the skin and then destroyed by actual cautery; while for the latter he would advise they be at once cut out with the knife. Ambrose Paré and Petit punctured them, squeezed out their contents and applied a bandage. Bayer tied the vein above and below before cutting; and Holmes, after dividing the skin, passed a thread under the vein and so tied the vessel leading from the varix towards the heart, under the impression that the blood, as it could no longer flow through the vessel leading from the varix, would stagnate and then coagulate. Capaletti employed galvano-puncture to induce coagulation of the blood in the varix.

The treatment today is the action of caustics applied over the course of the vessel; the subcutaneous division of the walls; or, by compressing it between a steel pin and a twisted suture. Brodie recommends that enough caustic should be used to insure the sloughing of the vessel, as a more certain mode of securing its destruction.

### VARICOSE ULCERS.

The varices in their constant work of interference by impeding the return of the blood, and by constantly producing habitual congestion, are rendered prone to ulceration, which gave rise to varicose ulcers and to the eczematous appearance of the surrounding surfaces. The ulcer generally appears to be deep, but it is only due to great amount of pain they produce. There are usually three or four in progress at one time, generally situated above the ankle or along the track of the tibia, and are oval in shape, indolent in their progress, and, as remarked before, they are painful at first. I have a patient now who complains if any of her family walk near the bed. She says the pain is so intense that she never expects to get better. This case is a good illustration of the ordinary manifestations of the disease. She had been troubled with varicose veins when pregnant, years ago, but she said they had all disappeared, and for years she never expected further trouble; when, all at once, a simple red spot appeared on her leg, which, in a few days had become an abscess. This was soon followed by three others going through the same process. She had to be kept under the influence of opiates for the first few days ; the local application of boracic acid was then discontinued, and I substituted a solution of cocaine and applied iodoform, iodol, and warm fomentations. Tr. ferri mur. is taken regularly and she now seems in a fair way to recover.

I mention this first case to illustrate the primary condition of varicose ulcers. But there is another form which I may call chronic, or recurrent, or of long standing, where the skin of the lower extremities seems to be permanently discolored, whether the varicose ulcer is present or not, and which is accompanied sometimes by venous hemorrhages or by ulcer. These are not so painful generally, but obstinate to the treatment. I wish to describe two cases of this type.

Case I.— Mrs. H—— came under observation with the condition as reported above. He had been going the rounds of the physician's offices like many cases of this class, which was somewhat peculiar on account of its indolence. Nothing seems to change the appearance of the ulcerated surface ; lead preparation, iodoform, boracic acid, and even caustics would irritate the surface without bringing any change. I gave her a mixture of Fowler's sol. and tinct. of iron, and by using cocaine hypodermically, I took up minute pieces of skin from the right arm and grafted the ulcer — two grafts at the first sitting only — using the absorbent cotton with iodoform and bandage to retain them in position. In a few days, to my satisfaction, there were signs of vitality ; the same process was repeated three times in succession, and in the space of six weeks the ulcer had completely healed.

Case II.— Mrs. S. —— came under my observation with the same history and the same symptoms, but one of the borders of the ulcer was more elevated, the color of the secretions of a greenish appearance and of an offensive odor. I first advised carbolic acid washes—iodoform and warm poultices—with the same mixture inwardly and in a few weeks, finding very little encouragement, I tried grafting ; but instead of improvement I irritated the ulcer so that I dared not repeat it. As a last resort I prescribed protoiodide of mercury, gr. one-sixth, twice a day, and gr. xx. of iod. potassium. Under this plan I had the pleasure of seeing a marked improvement, and it told its own story.

473 VIRGINIA STREET.

DISCUSSION.

Dr. THOS. LOTHROP: In treating varicose veins, I always tie off the vein and get good results. In varicose ulcers the results are unfavorable, as they occur in old, broken-down multiparous women. In such, I generally use the elastic bandage or rubber stocking.

Dr. E. CLARK: In treating this affection I elevate the limb, strap the edges of the ulcer, and cauterize. New granulations will be set up in a short time.

Dr. A. E. PERSONS: To heal a varicose ulcer, we must support the venous circulation. I generally use the adhesive straps; they equalize the circulation and stimulate granulations. Afterward the flannel bandage is employed.

Dr. DORR: When the patient coöperates, I generally get good results. Wash the ulcer with campho-phenique and carbolic acid, one dram each to one pint of water. I think the flannel bandage is superior to the rubber stocking.

Dr. FELL: In all these cases there is some devitalization of tissue, and we must overcome this obstacle. I believe that gravity has much to do with the formation of these ulcers.

Dr. BRIGGS: When I was a student, the injection of iodine was in vogue. I have had as many as twenty patients with varicose ulcers under treatment at the same time. I have always had them in old multiparous women of German birth. My treatment is as follows: I bandage the limb with flannel cut on the bias, and place a piece of linen soaked in carbolic acid over the ulcer. Calvert's carbolic acid I believe is almost a specific for this trouble. I generally use two ounces of the acid to two pints of the water.

Dr. E. A. SMITH: I have seen large sized veins in a young man and believe hereditary weakness plays an important rôle.

Dr. MYNTER's treatment is as follows: He freshens the edges, and applies skin grafts. Then places the limb in plaster. In severe cases he dissects out the veins.

---

"MEDICAL science has made such a progress," said the doctor, when speaking of his profession, "that it is impossible for anybody to be buried alive now." Then he wondered why everybody laughed.—*Boston Courier.*

